# Highlights, predictions, and changes

**DOI:** 10.1186/1742-4690-9-96

**Published:** 2012-11-15

**Authors:** Kuan-Teh Jeang

**Affiliations:** 1 The National Institutes of Health, Bethesda, MD, USA

## Abstract

Recent literature highlights at *Retrovirology* are described. Predictions are made regarding “hot” retrovirology research trends for the coming year based on recent journal access statistics. Changes in *Retrovirology* editor and the frequency of the *Retrovirology* Prize are announced.

## 

I recently wrote an editorial in *Cell and Bioscience* on the use of a novel algorithm to predict the future H-index of a scientist and his/her likelihood of “success” [1]. In a similar vein, as we approach the end of a calendar year, I examined access statistics of recently published *Retrovirology* papers to predict areas of highlighted interest for the coming year.

In parsing access frequencies over the preceding 12 months to recent *Retrovirology* papers, a few trends stood out. First, a disproportionately large number of highly accessed papers focused on cellular restriction factors and their activities on HIV-1 [2-5], and in particular on the newly discovered and characterized SAMHD1 protein [6,7]. Second, papers on cellular innate immunity to retrovirus infection also captured interest [8,9]. Third, the topic of nuclear import of HIV-1 pre-integration complex remains popular [10,11]. Lastly, a paper published in *Retrovirology* only 10 months ago on microRNA changes in HIV-1 infected individuals [12] remarkably has elicited more than 4,400 accesses already, suggesting significant timely and topical interest. Twelve months from now, I will revisit these papers and their research areas to check how their citation frequencies bear out the interest reflected by their access frequencies.

This year marks a first change in *Retrovirology* editors. Michael Lairmore, an editor of *Retrovirology* since its inception in 2004, assumed the Deanship of the Veterinary College at the University of California, Davis. His new academic responsibilities precluded his continued editing duties with *Retrovirology*. With Michael Lairmore’s departure, we welcome Persephone (Seph) Borrow of Oxford University as a new editor of *Retrovirology*. Seph brings to us added expertise in the immunology of retroviruses, and the journal will look to her leadership in expanding the publishing of papers in this research area.

*Retrovirology* is also making a change in the frequency of the *Retrovirology* Prize, which was awarded to Masao Matsuoka of Kyoto University in 2011 [13]. To date, the *Retrovirology* Prize has been awarded annually. However, going forward, with a view towards increasing the selection stringency of our prize winners, the editors have decided to award this Prize on a biannual basis. The aim is to award the *Retrovirology* Prize in the same year as and at our biannual *Frontiers of Retrovirology* meeting ( http://www.frontiers-of-retrovirology.com/). The rules for nomination and candidacy of the *Retrovirology* Prize remain the same, except that there will no longer be a distinction made between HIV vs. non-HIV virologists. With this editorial, we invite nominations for the 2013 *Retrovirology* Prize, which will be awarded at the *Frontiers of Retrovirology* meeting September 16–18 at Churchill College, Cambridge University, England.
